# Extent, nature and hospital costs of fireworks-related injuries during the Wednesday Eve Festival in Iran

**DOI:** 10.5249/jivr.v5i1.146

**Published:** 2013-01

**Authors:** Siros Alinia, Satar Rezaei, Rajabali Daroudi, Mashyaneh Hadadi, Ali Akbari Sari

**Affiliations:** ^a^Department of Health Economics and Management, School of Public Health, Tehran University of Medical Sciences, Tehran, Iran.; ^*b*^Injury Prevention and Safety Promotion Department, Ministry of Health, Tehran, Iran.; ^*c*^Knowledge Utilization Research Center, Tehran University of Medical Sciences, Tehran, Iran.

**Keywords:** Fireworks, Chaharshanbe Soori Injury, Health care costs, Injury prevention

## Abstract

**Background:**

Fireworks are commonly used in local and national celebrations. The aim of this study is to explore the extent, nature and hospital costs of injuries related to the Persian Wednesday Eve festival in Iran.

**Methods:**

Data for injuries caused by fireworks during the 2009 Persian Wednesday Eve festival were collected from the national Ministry of Health database. Injuries were divided into nine groups and the average and total hospital costs were estimated for each group. The cost of care for patients with burns was estimated by reviewing a sample of 100 patients randomly selected from a large burn center in Tehran. Other costs were estimated by conducting semi structured interviews with expert managers at two large government hospitals.

**Results:**

1817 people were injured by fireworks during the 2009 Wednesday Eve festival. The most frequently injured sites were the hand (43.3%), eye (24.5%) and face (13.2%), and the most common types of injury were burns (39.9%), contusions/abrasions (24.6%) and lacerations (12.7%). The mean length of hospital stay was 8.15 days for patients with burns, 10.7 days for those with amputations, and 3 days for those with other types of injury. The total hospital cost of injuries was US$ 284 000 and the average cost per injury was US$ 156. The total hospital cost of patients with amputations was US$ 48 598. Most of the costs were related to burns (56.6%) followed by amputations (12.2%).

**Conclusions:**

Injuries related to the Persian Wednesday Eve festival are common and lead to extensive morbidity and medical costs.

## Introduction

Fireworks are widely used in national and cultural celebrations around the world. The Hari Raya Festival in Malaysia, Independence Day in the United States and the Last Wednesday Eve festival known as Chaharshanbe Soori in Iran are some examples.^1-3^ Chaharshanbe Soori is an annual cultural celebration held on the evening before the last Wednesday of the Persian calendar year. It was originally celebrated with small, simple fireworks, but recently it has become characterized by the widespread use of many illegal and homemade explosive devices.^4^ This has resulted in severe injuries to people and extensive property damage. One recent study noted that, “every year in Iran many individuals, mainly children and young adults, are injured by various fireworks ranging from firecrackers to homemade grenades”.^1^

These injuries have given rise to extensive concerns about the safety of fireworks in Chaharshanbe Soori ceremonie.^5^ In the United States, fireworks annually account for approximately 9800 injuries requiring treatment in an emergency department.^3^ There were 2500 structural and vehicle fires associated with fireworks in the United States in 2005, causing an estimated US$ 39 million in direct property damage.^6^ As a result, policies have been developed to reduce such injuries. These policies involve investments in systems and programs designed to monitor and learn from fireworks-related injuries. In Iran, the Ministry of Health developed a National System called the Safe Community Project (SCP), established in 2003. 

Few studies have evaluated the extent, nature and medical costs associated with injuries during national and cultural celebrations in Iran. ^1,7-10^ To our knowledge only one such study estimated the medical costs of injuries during the Persian Wednesday Eve festival in Iran, but that study was mainly based on sample data collected retrospectively via interviews with a random sample of families in a single city.^9^ Previous studies, moreover, did not carefully document the nature, costs and consequences of injuries associated with fireworks in Iran. In this article we report the results of a systematic study of the hospital costs of Chaharshanbe Soori fireworks-related injuries in Iran. We used data compulsorily reported to the national database specially designed for such injuries at the Iran Ministry of Health to estimate the extent, nature and hospital costs associated with these injuries. This source of data can be assumed to be more accurate and reliable than sources used for previous analyses.

## Methods

The data reported to the SCP database for injuries related to fireworks in 2009 were obtained from Iranian Ministry of Health. The aim of the SCP database is to collate reports of injuries caused by fireworks during the Chaharshanbe Soori festival so that the Ministry can determine the characteristics of these injuries with a view to developing preventive measures. It collects data on the number of patients injured, each patient’s age, sex and sociodemographic status, the site, type, severity and outcome of the injury, the type of firework involved, and the date and place of the incident. The data are collected on a structured form at hospitals and healthcare centers where patients receive treatment, and then sent to the Ministry via standardized software.

No data about the cost of treatment for each patient are available in the SCP database. We used the SCP data for different types of injury to classify patients into nine groups: contusions/abrasions, lacerations, wounds, bone injury, amputation, first degree burns, second degree burns, third degree burns and other injuries (e.g. loss of sight). Then the SCP data for severity of the injuries were used to stratify patients with each type of injury into three subgroups representing outpatient, inpatient and transfer to specialty hospital, making a total of 27 groups. The number of patients in each group was known, but because data about treatment costs were not available in the SCP database, we used the following model to estimate the average cost of treatment for each study group: total cost = ∑ (number of patients in each group × average cost per patient in that group). 

To estimate the average cost of treatment per patient in each injury category we used the following approach: Most injuries were burns, so to estimate the average cost per patient with burns a sample of 100 patients were randomly selected from a large burn center in Tehran. The average costs were discussed with and verified by the hospital managers. To estimate the average cost per patient in the other injury groups, semistructured interviews were conducted with expert managers at two large government hospitals. During interviews conducted for each injury group, we collected data on the average cost per outpatient treatment, average length of hospital stay, average cost per day, and the total cost associated with each type of injury.

The data were entered into Excel software for analysis. The results are reported as the number and percentage of each type of injury and each level of severity according to the age and sex of patients and site of injury in the body. Finally the cost of injury was estimated for each type of injury and for the total study sample.

## Results

**Types of injury**

A total of 1817 injuries related to Chaharshanbeh Soori fireworks were reported to the national SCP database in 2009. Most patients (1512, 83.2%) were males. About one fourth of the injuries (24.8%) occurred in individuals between the ages of 15 and 20 years, and about one fifth (20.5%) involved people aged 20 to 25 years (Table 1).

**Table:** Table 1:**Number and percent of individuals injured by fireworks during the Persian Wednesday Eve festival by age group.**

Age group (years)	Number	Percent
<10	184	10.0
10-15	302	16.6
15-20	469	25.8
20-25	373	20.5
25-30	188	(10.3
30-35	103	5.7
>35	187	10.4
Unknown	11	0.6
Total	1817	100

Most injuries (80.2%) occurred in streets, followed by homes (13%) and other place such as parks (6.8%). Hand injuries were the most common site of injury (786 patients, 43.3%) followed by eye (445, 24.5%) and face injuries (240, 13.2%) (Table 2).

**Table:** Table 2:**Number and percentage of fireworks-related injuries during the Persian Wednesday Eve festival by site of injury.**

Injured part	Number	Percent
Hand	786	43.3
Eye	445	24.5
Face	240	13.2
Leg	138	7.55
Other parts	208	11.45
Total	1817)	100

Firecrackers (60.7%) and homemade grenades (13.75%) were the devices that most frequently caused injuries, followed by fire, fountain and sparklers (Table 3). Users accounted for almost 61% of all people injured, signiﬁcantly outnumbering bystanders and others (Table 4).

**Table:** Table 3:**Number and percentage of fireworks-related injuries during the Persian Wednesday Eve festival by types of device.**

Injured part	Number of injuries	Percent of injuries
Firecracker	1103	60.7
Homemade grenade	249	13.75
Fire	135	7.45
Fountains and sparklers	121	6.7
Others	209	11.4
Total	1817	100

**Table:** Table 4:**Number and percentage of fireworks-related injuries during the Persian Wednesday Eve festival by firework-user status.**

Firework user status	Number of injuries	Percent of injuries
User	1107	60.92
Bystander	440	24.2
Uncertain	270	14.88
Total	1817	100

Burns (39.9%), abrasions and contusions (24.6%) and lacerations (12.7%) were the most common types of injury (Table 5). Most patients with burns (85%) were treated as outpatients. Of all burn injuries, first degree burns were the most frequent (457, 63.2%), followed by second degree (241, 33.3%) and third degree burns (25, 3.5%). Most injured patients (82.08%) were treated as outpatients, although 17.92% were hospitalized, of whom 40% were transferred to a specialty hospital to receive additional care (Table 6).

**Table:** Table 5:**Number and percent of fireworks-related injuries during the Persian Wednesday Eve festival by site of injury.**

Injury type	Number	Percent
Burns	723	39.8
Contusion/Abrasion	447	24.6
Laceration	230	12.6
Wound	194	10.63
Bone injuries	29	1.6
Amputation	14	0.77
Other case	180	10
Total	1817	100

**Table:** Table 6:**Number and percent of fireworks-related injuries during the Persian Wednesday Eve festival by type of treatment.**

Type of injury	Type of treatment			Total Number (%)
	Outpatient Number (%)	Inpatient Number (%)	Transfer to specialty hospital Number (%)	
Contusion/abrasion	413 (22.7)	16 (0.88)	18 (0.99)	447 (24.6)
Laceration	153 (8.46)	60 (3.3)	17 (0.94)	230 (12.66)
Wound	177 (9.74)	8 (0.44)	9 (0.5)	194 (10.68)
Bone injury	14 (0.77)	10 (0.55)	5 (0.28)	29 (1.596)
Burn I	417 (22.9)	22 (1.21)	18 (0.99)	457 (25.15)
Burn II	185 (10.2)	42 (2.31)	14 (0.77)	241 (13.26)
Burn III	14 (0.77)	6 (0.33)	5 (0.28)	25 (1.376)
Amputation	0 (0)	3 (0.17)	11 (0.61)	14 (0.77)
Other	118 (6.49)	26 (1.43)	36 (1.98)	180 (9.906)
Total	1491 (82.08)	193 (10.63)	133 (7.32)	1817 (100)

Three patients (1.65 per 1000) died and four were permanently disabled (2.2 per 1000) because of their injuries.

**Hospital costs of injuries**

The average cost of hospital outpatient treatment for patients with burns was US$ 10.32. The mean length of hospital stay was 4.7 days for first degree burns, 9 days for second degree burns and 16 days for third degree burns. The average hospital cost per patient was US$ 589 for first degree, US$ 1475.78 for second degree and US$ 4331.86 for third degree burns. The total cost of treating burn patients was US$ 160 908.73, and hospitalized patients accounted for approximately 96% of this figure. 

The average length of hospital stay was 10.7 days for patients with amputations and 3 days for other types of injury including lacerations, wounds and contusions/abrasions. The average cost of treatment per patient was US$ 48 597.89 for amputations and US$ 3471.27 for other types of injury including lacerations, wounds and contusions/abrasions. The total hospital costs of injuries related to the 2009 Persian Wdnesday Eve festival was US$ 283 880, and the average cost of each injury was US$ 156.23. Slightly more than half of all hospital costs (56.6%) were incurred by patients with burns, and 17.2% were related with injuries that led to amputation (Table 7).

**Table:** Table 7:**Costs associated with fireworks-related injuries during the Persian Wednesday Eve festival.**

Type of injury	Cost (US$)	Percentage of total cost
Burn	160 908.74	56.6
Amputation	48 597.90	17.2
Contusion/abrasion	13 923.70	5
Laceration	13 198.68	4.6
Wound	12 907.90	4.5
Bone injury	6194.73	2.1
Other	28 149.26	10
Total cost	283 880.89	100

## Discussion

Fireworks are used in local and national celebrations across the world including in the US, UK, China, India, Libya and Iran.^11-14^ This study showed that 1817 people were injured by fireworks during the Chaharshanbe Soori festival in Iran in 2009. Most injured people were males (83.2%) and most injuries occurred in the 15-to-20 year age group. These findings are consistent with studies in other countries that showed males to be a high-risk group for such injuries.^7,15,16^ A study in Greece showed that 70% of such injuries occurred in boys between 10 and 14 years of age.^17^

In our study sample the hand (43.3%), eye (24.5%) and face (13.2%) were the most frequently injured sites, which is consistent with the findings of other studies.^8,16,18-21,22^ Burns (39.9%) contusions/abrasions (24.6%) and lacerations (12.7%) were the most common types of injury in the present study. Other studies also showed that burns and lacerations were the most common types of injury.^7,8,21-23,24^ According to our data, about 82% of injured patients were treated as outpatients and approximately 18% were hospitalized, which is in line with the results of similar studies.^20,21^ Witsaman et al. (2006) found that in the USA, 91.6% of children referred to hospitals for fireworks-related injuries were treated in the emergency department and discharged without inpatient hospitalization, whereas 5.3% were hospitalized and 2.3% were transferred to another institution. For the remaining 0.8% the disposition was other or unknown.^21^

We found that the mean length of hospital stay was 8.15 days for patients with burns, 10.7 days for those with amputations, and 3 days for patients with other types injury. Aghakhani et al. reviewed 639 hospitalized patients with burns and reported that the average length of hospital stay was 7.76 days.^25^ Kabirzade et al. found that the average length of hospital stay was 6 days for these patients,^26^ and Khorasani et al. reported an average length of hospital stay of 21.6±11.2 days for a sample of 113 patients with burns.^27^ It seems that in general, the burns in the small sample of patients studied by Khorasani and colleagues were more severe, and that the standard deviation was relatively large. 

The total hospital cost of Chaharshanbe Soori fireworks-related injuries in 2009 was about US$ 284 000, and average cost per patient was about US$ 156. The total hospital cost for patients with amputations was US$ 48 598. Most hospital costs were related to treatment for burns (56.6%) and amputations (12.2%). The average cost per burned patient of patients hospitalized with Chaharshanbe Soori fireworks-related injuries was about US$ 1440, and the cost for patients with second and third degree burns was about US$ 2904. These findings are similar to the results of a previous study conducted in Iran,^27^ which reported a mean cost per patient of US$ 2666.6±21 for second and third degree burn.^27^ The high figures reflect the fact that patients with burns normally need a relatively long hospital stay, expensive antibiotics and surgery. Despite the small number of patients with amputations, the total cost of care for this subgroup was high because avulsion and amputation normal require prolonged hospitalization and expensive surgeries.

In a study carried out in Iran in 2007, mean household fireworks-related expenditures were US$ 37 for property damage and US$ 513 for medical care and related expenses.^9^ In the present analysis we estimated only direct medical care costs based on public hospital tariffs. We are aware that a small proportion of patients may have been treated at private hospitals, which are more expensive than public hospitals, so we may have underestimated the overall costs for all persons injured by fireworks.

## Conclusion

This study shows that injuries related to the Persian Wednesday Eve festival are common, lead to considerable morbidity, and result in a large drain on Iran’s healthcare resources. Efforts should be directed to reducing these injuries and mitigating their consequences. To prevent fireworks- related injuries and contain the associated costs, we recommend the following strategies: first, media campaigns to increase public awareness about fireworks-related injuries; second, stricter industry standards for firecracker safety; and third, enforcement of a minimum legal age of 18 years for purchasing fireworks.

## References

[1] Saadat S, Naseripour M, Rahimi B (2009). Safety preparedness of urban community for New Year fireworks in Tehran. Burns.

[2] Isa AR, Moe H (1991). Fireworks related injuries during Hari Raya festival in Hospital Universiti Sains Malaysia-- 1986 to 1990. Med J Malaysia.

[3] Greene MA, Joholske J. Fireworks Annual Report: fireworks-related deaths, emergenc department-treated injuries, and enforcement activities during 2007. Washington, DC: Consumer Product Safety Commission, 2008.

[4] Mohammadi SF, Mohammadi SM, Ashrafi E, Hatef E, Rahbari H (2011). Chaharshanbe -Soori fireworks and public health. Iranian Journal of Ophthalmology.

[5] Peden M, Oyegbite K, Ozanne-Smith J, Haydar AA, Branche C, Rahman AF, et al. World report on child injury prevention. Geneva: World Health Organization, 2008: 86. 26269872

[6] Hall JR. Fireworks. National Fire Protection Association, Fire Analysis and Research Division 2008, http://www.nfpa.org/assets/files/pdf/os.fire works.pdf, accessed 29 August 2008.

[7] Mansouri MR, Mohammadi SF, Hatef E, Rahbari H, Khazanehdari MS, Zandi P (2007). The Persian Wednesday Eve Festival "Charshanbe-Soori" fireworks eye injuries: a case series. Ophthalmic Epidemiol.

[8] Tavakoli H, Khashayar P, Amoli HA, Esfandiari K, Ashegh H, Rezaii J (2011). Firework-related injuries in Tehran's Persian Wednesday Eve Festival (Chaharshanbe Soori). J Emerg Med.

[9] Saadat S, Naseripour M, Smith GA (2010). The health and economic impact of fireworks-related injuries in Iran: a household survey following the New Year’s Festival in Tehran. Injury.

[10] Farasat Kish R, Molla Sadeghi Gh.A, Ansari H (2001). Burns of the firework wednesday celebration in Iranian years 1998 and 1999 on patients who referred to hospitals of Iran University of medical Sciences. Journal of Legal Medicine of Islamic Republic of Iran.

[11] American Academy of Pediatrics: Committee on Injury and Poison Prevention. Fireworks-related injuries to children. Pediatrics.2001Jul;108(1):190-1. 10.1542/peds.108.1.190 11433076

[12] Fogarty BJ, Gordon DJ (1999). Firework related injury and legislation: the epidemiology of firework injuries and the effect of legislation in Northern Ireland.. Burns.

[13] Jing Y, Yi-qiao X, Yan-ning Y, Ming A, An-huai Y, Lian-hong Z (2010). Clinical analysis of firework-related ocular injuries during Spring Festival 2009. Graefes Arch Clin Exp Ophthalmol.

[14] Dhir SP, Shishko MN, Krewi A, Mabruka S (1991). Ocular fireworks injuries in children. J Pediatr Ophthalmol Strabismus.

[15] Zohar Z, Waksman I, Stolero J, Volpin G, Sacagiu E, Eytan A. Injury from fireworks and firecrackers during holidays [Hebrew]. Harefuah. 2004 Oct;143(10):698-701, 768. 15521342

[16] Centers for Disease Control and Prevention (CDC). Injuries from fireworks in the United States. MMWR Morb Mortal Wkly Rep. 2000 Jun 23; 49(24):545-6. 10923857

[17] Vassilia K, Eleni P, Dimitrios T (2004). Firework-related childhood injuries in Greece: a national problem.. Burns.

[18] Foged T, Lauritsen J, Ipsen T (2007). Firework injuries in Denmark in the period 1995/1996 to 2006/2007. Ugeskr Laeger.

[19] See LC, Lo SK (1994). Epidemiology of fireworks injuries: the National Electronic Injury Surveillance System, 1980–1989. Ann Emerg Med.

[20] Sheller JP, Muchardt O, Jonsson B, Mikkelsen MB (1995). Burn injuries caused by fireworks: effect of prophylaxis. Burns.

[21] Witsaman RJ, Comstock RD, Smith GA (2006). Pediatric fireworks-related injuries in the United States: 1990–2003. Pediatrics.

[22] Mutto M, Lawoko S, Nansamba C, Ovuga E, Svanstrom L (2011). Unintentional childhood injury patterns, odds, and outcomes in Kampala City: an analysis of surveillance data from the National Pediatric Emergency Unit. J Inj Violence Res.

[23] D’Ippolito A, Collins CL, Comstock RD (2010). Epidemiology of pediatric holiday-related injuries presenting to US emergency departments. Pediatrics.

[24] Toon MH, Maybauer DM, Arceneaux LL, Fraser JF, Meyer W, Runge A (2011). Children with burn injuries--assessment of trauma, neglect, violence and abuse. J Inj Violence Res.

[25] Aghakhani N, Rahbar N, Feizi A, Karimi H, Vafa Shoar N (2008). Epidemiology of hospitalized patients in burn ward of Imam Khomeini Hospital in Urmia (2005). Behbood, The Scientific Quarterly.

[26] Kabirzadeh A, Zamani Kiyasari A, Bagherian Farahabadi E, Mohseni Saravi B, Kabirzadeh A, Tavasoli Ashrafi A (2007). Burn death rate among hospitalized patients in Zare teaching hospital of Mazandaran medical University, Sari, Iran (2002-04). Journal of Gorgan University of Medical Sciences.

[27] Khorasani Gh, Salehifar E, Eslami G (2007). Causes of burns and their outcomes in patients hospitalized in the burn division of zare hospital 2006-2007. Journal of Mazandaran University of Medical Sciences.

